# H_2_O_2_ biosensor consisted of hemoglobin-DNA conjugate on nanoporous gold thin film electrode with electrochemical signal enhancement

**DOI:** 10.1186/s40580-018-0172-z

**Published:** 2019-01-03

**Authors:** Jinhee Jo, Jinho Yoon, Taek Lee, Hyeon-Yeol Cho, Ji-Young Lee, Jeong-Woo Choi

**Affiliations:** 1 Department of Chemical and Biomolecular Engineering, Sogang University, 35 Baekbeom-ro (Sinsu-dong), Mapo-gu, Seoul, 121-742 Republic of Korea; 2 Department of Chemical Engineering, Kwangwoon University, 20 Kwangwoon-ro, Nowon-gu, Seoul, 01897 Republic of Korea; 3 Department of Chemistry and Chemical Biology, Rutgers, The State University of New Jersey, Piscataway, NJ 08854 USA

**Keywords:** Biosensor, DNA hybridization, Hemoglobin, Hydrogen peroxide, Nanoporous gold thin film

## Abstract

In this research, we developed electrochemical biosensor which was composed of hemoglobin (Hb)-DNA conjugate on nanoporous gold thin film (NPGF) for hydrogen peroxide (H_2_O_2_) detection. For the first time, Hb and DNA was conjugated as a sensing platform for uniform orientation of Hb on electrode. The newly developed Hb-DNA conjugate was designed to prevent Hb from aggregation on electrode. DNA hybridization of Hb-DNA conjugate and complementary DNA (cDNA) on NPGF electrode induced uniformly assembled biosensor. Furthermore, NPGF electrode fabrication method was introduced to the increment of the surface area. To confirm the conjugation of Hb-DNA conjugate, sodium dodecyl sulfate polyacrylamide gel electrophoresis (SDS-PAGE) and ultraviolet–visible spectroscopy (UV–VIS) were used. Formation of the NPGF electrode was verified by scanning electron microscope (SEM). Atomic force microscopy (AFM) was operated for the confirmation of Hb-DNA immobilization on electrode. The electrochemical property of fabricated electrode was investigated by cyclic voltammetry (CV). Also, H_2_O_2_ sensing performance of fabricated electrode was investigated by amperometric i-t curve technique. This sensor showed a wide linear range from 0.00025 to 5.00 mM and a correlation coefficient of R^2^ = 0.9986. The detection limit was 250 nM. Proposed biosensor can be utilized as a sensing platform for development of biosensor.

**Electronic supplementary material:**

The online version of this article (10.1186/s40580-018-0172-z) contains supplementary material, which is available to authorized users.

## Introduction

Over the past decades, various researches for the development of biosensors have received the huge interests for biomedical and environmental applications. Several biomolecules such as protein and DNA have some advantages for the development of biosensors including fast response reaction and remarkable selectivity [1, 2]. Particularly, metalloproteins have been evaluated as suitable materials for biosensors because of the direct redox properties and the fast electrochemical response [3–5]. Chen’s group used glucose oxidase as metalloprotein for detecting trichloroacetic acid for glucose detection [6]. Sun’s group used myoglobin as metalloprotein for detecting trichloroacetic acid [7]. Also, our group especially developed various biosensors using metalloprotein [8–10].

Hydrogen peroxide (H_2_O_2_) is widely known as unstable and reactive product in biological system which affected harmful effect to living cells. As a main by-product of enzymatic reactions, concentration of H_2_O_2_ is considered as a parameter for activity coefficient of physiological reactions [11]. Consequently, various types of biosensors have been developed to detect H_2_O_2_ [12, 13]. Kauffmann’s group developed the H_2_O_2_ biosensor using enzyme horseradish peroxidase (HRP) entrapped in a polypyrrole electrode [14]. Luo’s group fabricated H_2_O_2_ biosensor which was based on cytochrome c [15].

However, conventional biosensors had some limitations such as high detection limit and irregularly assembled biomolecules on the electrode which interrupted the accurate interaction between the target material and sensing molecule (Additional file 1: Table S1). To overcome these limitations, the expansion of surface area of the electrode and the uniform immobilization of the sensing molecule on the electrode could be expected as proper solution [16]. To expand the surface area of the electrode, the nanoporous gold thin film (NPGF) was widely used for electrode preparation [17]. The electrochemical signal of biomolecules could be enhanced due to the immobilization of more sensing molecules on the electrode by the surface expansion and the enhanced electron transfer rate [18].

DNA has the unique property that single strand DNA can form the hybridized double strand DNA with high selectivity by introduction of complementary DNA (cDNA). Thus, DNA has been widely used to fabricate the self-assembled structure precisely [19, 20]. This unique property of DNA can be applied to fabricate the well orientated biomolecule layer on the electrode. The NPGF electrode technique and uniform orientation via DNA hybridization showed synergy effect through electrochemical signal enhancement.

In this point of view, for the first time, an electrochemical biosensor composed of hemoglobin (Hb)-DNA conjugate on the NPGF electrode was developed to detect H_2_O_2_ with electrochemical signal enhancement and high selectivity. Newly developed Hb-DNA conjugate was used as a sensing platform. Hb and DNA were conjugated by sulfosuccinimidyl 4-cyclohexane-1-carboxylate (Sulfo-SMCC). Also, the electrodeposition technique was applied to fabricate the NPGF electrode for the expansion of the surface area of electrode. The thiol-modified cDNA was directly immobilized on the NPGF electrode by self-assembly of gold-thiol interaction. Then, as a biomolecular probe, Hb-DNA conjugate was immobilized on the electrode by DNA hybridization. Hb is the metalloprotein with four iron ions in its core which can detect H_2_O_2_ by the electrochemical reduction reaction with H_2_O_2_ accompanying the unique redox properties. Also, Hb can detect H_2_O_2_ by the electrochemical reduction reaction with H_2_O_2_. As Hb is one kind of heme proteins, it contains heme groups. Iron in the heme can undergo oxidation and reduction over a wide range of potentials around heme groups. Because of the redox property of heme proteins, they have the great potential to be applied for biosensors. The final goal of this research was uniform orientation of protein to avoid aggregation. Consequently, well orientated protein without aggregation was expected to show enhanced performance comparing conventional self-assembled sensors.

**Fig. 1:**
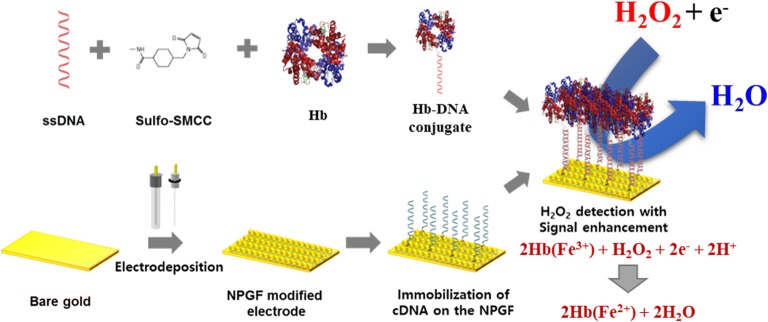
The schematic diagram of Hb-DNA conjugate on the NPGF electrode

Fabrication of the NPGF electrode was investigated by scanning electron microscope (SEM) and atomic force microscopy (AFM). To confirm the fabrication of Hb-DNA conjugate, sodium dodecyl sulfate polyacrylamide gel electrophoresis (SDS-PAGE) and ultraviolet–visible spectroscopy (UV–VIS) were used. Cyclic voltammetry (CV) was used for the electrochemical signal enhancement of fabricated biosensor. Also, immobilization of Hb-DNA conjugate on the electrode was investigated by AFM. Finally, H_2_O_2_ sensing performance of fabricated biosensor was estimated by amperometric i–t curve technique. Figure 1 showed the schematic diagram of fabricated Hb-DNA conjugate on the NPGF electrode via DNA hybridization with uniform orientation.

## Materials and methods

### Materials

Hemoglobin from bovine blood, gold chloride trihydrate 99.9% trace metals basis, L-ascorbic acid (AA), uric acid (UA), sodium nitrite (NaNO_2_), sodium bicarbonate (NaHCO_3_) solution and human serum from human male AB plasma were purchased from Sigma-Aldrich (USA). Polyethylene glycol (PEG) was purchased from Yakuri Pure Chemicals Co. LTD. (Japan). To conjugate Single stranded DNA (ssDNA) to the amide end of hemoglobin, the 5 prime end of ssDNA (5′-ATAAAAAAAGCGCGGGGGTTCCGCG-3′) was modified with a thiol group. The 5 prime end of cDNA (5′-AAAATAAAAACGCGCGGAACCCCCGCGC-3′) was also modified with a thiol group to immobilize on the NPGF electrode. ssDNA and cDNA were synthesized by Bioneer (South Korea). Sulfo-SMCC, and Bond-Breaker™ tris(2-carboxyethyl)phosphine (TCEP) solution were purchased from Thermo Fisher Scientific (USA). Gold substrate was purchased from National Nanofab Center (South Korea). Precision Plus Protein™ Unstained Protein Standards was purchased from Bio-rad (USA). Ethyl alcohol anhydrous, sulfuric acid, and H_2_O_2_ solution were purchased from Daejung Chemical (South Korea).

### Fabrication of the NPGF electrode

First of all, the electrodes were cleaned using a piranha solution (H_2_SO_4_: H_2_O_2_, 8:2 v/v) for 5 min, then deionized water (DIW) was used to wash electrode, and N_2_ gas was used to dry the substrate. According to the strong oxidizing property of the piranha solution, it could remove organic materials while making the surface highly hydrophilic.

To fabricate uniformly electrodeposited NPGF electrode, 1 mL of 10 mM gold chloride trihydrate aqueous solution and 4 mL of PEG aqueous solution (20 μL per 1 mL of DIW) were mixed. The three electrode system was utilized to fabricate the NPGF electrode by electrodeposition. Our three electrode system is composed as it follows, cleaned gold electrode was connected to working electrode, platinum wire was used as counter electrode and silver/silver chloride (Ag/AgCl) double-junction electrode was used as a reference electrode. And all of electrode supplies were purchased from CH Instruments (CHI). Under the room temperature, electrodeposition technique was performed at − 1.3 V for 30 s with multi-potential steps technique using potentiostat (CHI-660A, CHI, USA). The formation of the surface was investigated by SEM (SUPRA 55VP, Carl Zeiss, Germany).

### Conjugation of ssDNA and Hb

To make Hb-DNA conjugate, Sulfo-SMCC was used as a crosslinker between Hb and ssDNA [21]. For preparation, ssDNA and Hb were dissolved in Tris–EDTA (TE) buffer at 10 μM concentration each. To fabricate Hb-ssDNA conjugate, 10 µL of 1.0 M dithiothreitol (DTT) was added to every 100 µL of ssDNA solution. Then, the solution was incubated for 15 min at room temperature. Consumed DTT was removed using pure ethyl acetate. After the removal of consumed DTT, 10 µL of Sulfo-SMCC (10 mM) was added immediately, because free sulfhydryl group becomes unstable after the removal of DTT. The mixture was incubated for 2 h at 4 °C. After incubation, 10 µM of hemoglobin solution was added as the same volume ratio and was incubated for 6 h at 4 °C. The thiol-modified ssDNA is reacted with Sulfo-SMCC. And then, the the amine group of Hb was added to thiol-modified ssDNA-Sulfo SMCC for form amide bond. Finally, Hb-SMCC-DNA conjugate was prepared [21].

### Immobilizing Hb-DNA conjugate on the NPGF

As the gold-thiol interaction was much stronger than dithiol interaction, thiol modified cDNA was immobilized on the NPGF electrode uniformly by self-assembly [22, 23]. As the DNA molecule has negative charge at pH 7.4, its repulsive force between DNA–DNA interactions induced uniform self-assembly on the gold substrate. 10 μM of cDNA was dropped on the NPGF electrode sufficiently and kept in 4 °C for 6 h. Then, N_2_ gas was used to dry the cDNA-immobilized NPGF electrode. Prepared Hb-DNA conjugate was immobilized on the electrode via DNA hybridization. To confirm the electrochemical signal enhancement of the proposed biosensor, only Hb immobilized electrode was prepared by the chemical linker 6-mercaptohexanoic acid (6-MHA) for comparison. To fabricate this, 6-MHA was immobilized on the electrode with self-assembly method for 3 h at 4 °C. Then after 3 h, 6-MHA solution was dried by N_2_ gas. Then, Hb was spread on to the electrode for another 3 h at 4 °C with self-assembly method.

### Electrochemical investigation of NPGF/cDNA/Hb-DNA conjugate biosensor

The electrochemical property of NPGF/cDNA/Hb-DNA conjugate biosensor was analyzed using an electrochemical workstation. As the applied parameters for CV, voltage range of 0.6 V to − 0.2 V, 50 mV/s scan rate and at 1 × 10^−5^ (A/V) sensitivity were used. And phosphate buffered saline (PBS) was used as an electrochemical buffer [24]. After CV investigation, amperometric i-t curve technique was performed to investigate the performance in H_2_O_2_ detection. Parameters for amperometric i–t investigation were − 0.3 V initial potential, 0.1 s sampling interval time and 2 × 10^−6^ (A/V) sensitivity.

## Results and discussion

### Verification of the NPGF electrode formation

**Fig. 2:**
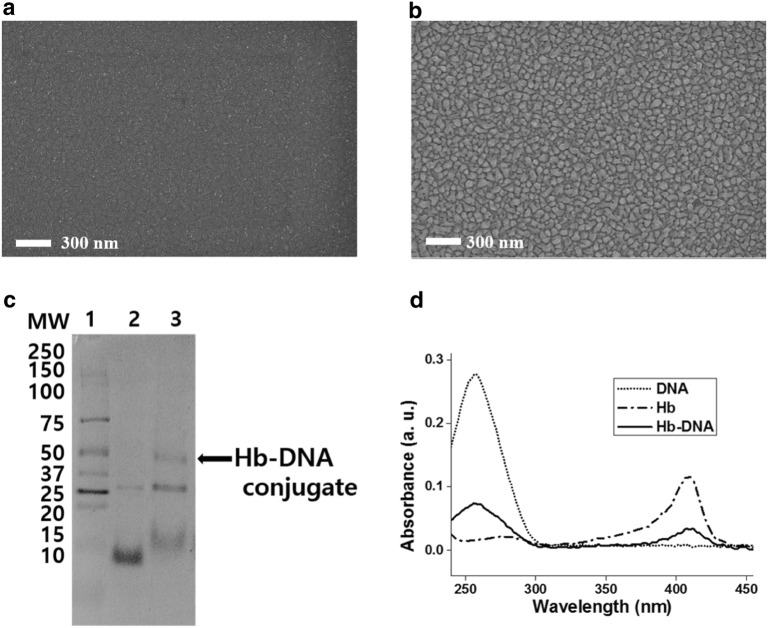
SEM images of **a** bare gold; **b** NPGF electrode; **c** SDS-PAGE (lane 1 for protein ladder, lane 2 for Hb, lane 3 for Hb-DNA conjugate); **d** UV–VIS results of Hb-DNA conjugate

Figure 2 showed the SEM results of the bare gold electrode and fabricated NPGF electrode. In Fig. 2a, the flat gold substrate was shown accompanying around 10 nm size of small clusters. By applying the − 1.3 V for 30 s on the bare gold electrode, gold nanoporous with diameter of 40.69 nm were uniformly deposited onto gold electrode in Fig. 2b and Additional file 1: Fig. S2. Through the NPGF electrode, surface extension of the substrate was investigated. Additional electrodeposition experiments were conducted to find optimized condition for the NPGF electrode. Additional file 1: Fig. S1 showed aggregation of gold nanoparticles with higher voltage or more time.

### Confirmation of Hb-DNA conjugate

In Fig. 2c, protein ladder and Hb are shown in Lane 1 and Lane 2, respectively. The Hb/Sulfo-SMCC/ssDNA conjugate is shown in Lane 3. In Lane 2, two bands (10 kDa for Hb, 25 kDa for aggregated Hb) were detected. In Lane 3, three bands (15 kDa for DNA, 25 kDa for aggregated Hb, and 40 kDa for DNA-aggregated Hb conjugate) were found. Therefore, Hb-DNA conjugate was successfully fabricated.

Figure 2d showed UV–VIS spectra of ssDNA, Hb and Hb-DNA conjugate. The ssDNA showed absorbance at 270 nm wavelength. This absorbance was attributed from the chromophoric groups in purine (adenine and guanine) and pyrimidine (cytosine and thymine) parts which are related to the electronic transition [25]. Also, Hb showed Soret absorption band of iron heme at 420 nm wavelength. This Soret peak could be shifted or disappeared if the Hb is denatured [26]. Consequently, the Hb-DNA conjugate showed absorbance at both of previous mentioned area (270 nm and 420 nm wavelength). Based on results of SDS-PAGE, Hb-DNA conjugate was confirmed at lane 3 showing 40 kDa of molecular weight. And Hb-DNA conjugate was also confirmed while having Hb and DNA UV–VIS peak at 270 nm, 420 nm respectively. We confirmed the successful fabrication of Hb-DNA conjugate by using Sulfo-SMCC.

### Morphology investigation of the fabricated biosensor

**Fig. 3:**
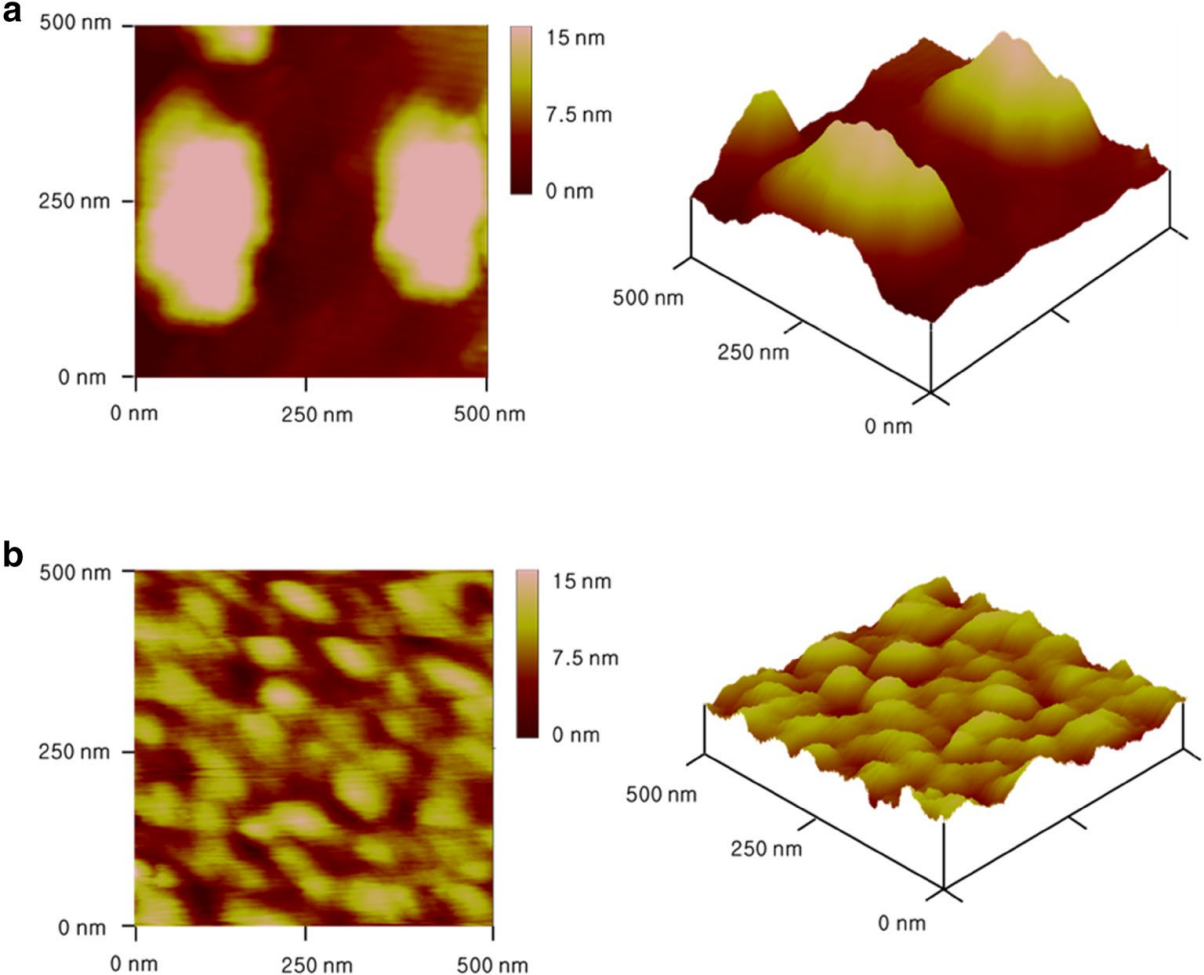
AFM results of **a** aggregated Hb/6-MHA/gold electrode; **b** Hb/DNA hybridization/gold electrode

AFM was utilized to examine the surface of the fabricated biosensor. As shown in Fig. 3a, Hb with chemical linker, 6-MHA, was aggregated on the surface of bare gold electrode with 200 nm size. And the surface of Hb via 6-MHA linker also showed root mean square (RMS) roughness 6.331 ± 1.293. On the other hand, Hb via DNA hybridization was uniformly immobilized onto the bare gold electrode without aggregation. We confirmed uniform immobilization through not only AFM data but also RMS roughness value. Consequently, RMS roughness showed 1.021 ± 0.481 which is diminished to one-sixth of previous RMS roughness of 6-MHA method [27, 28]. The 2D and 3D morphologies of the fabricated electrodes are shown in Fig. 3a, b, respectively. Chemically linked Hb showed six times higher roughness value than DNA-hybridization method (Table 1).

**Table 1:** Analysis of surface roughness

	6-MHA/Hb	DNA/Hb
Roughness average (RA) (nm)	5.687 ± 1.189	0.848 ± 0.416
RMS roughness (Rq) (nm)	6.331 ± 1.293	1.021 ± 0.481
Maximum roughness (Rmax) (nm)	17.803 ± 3.488	4.019 ± 1.749

### Electrochemical investigation of the fabricated biosensor

**Fig. 4:**
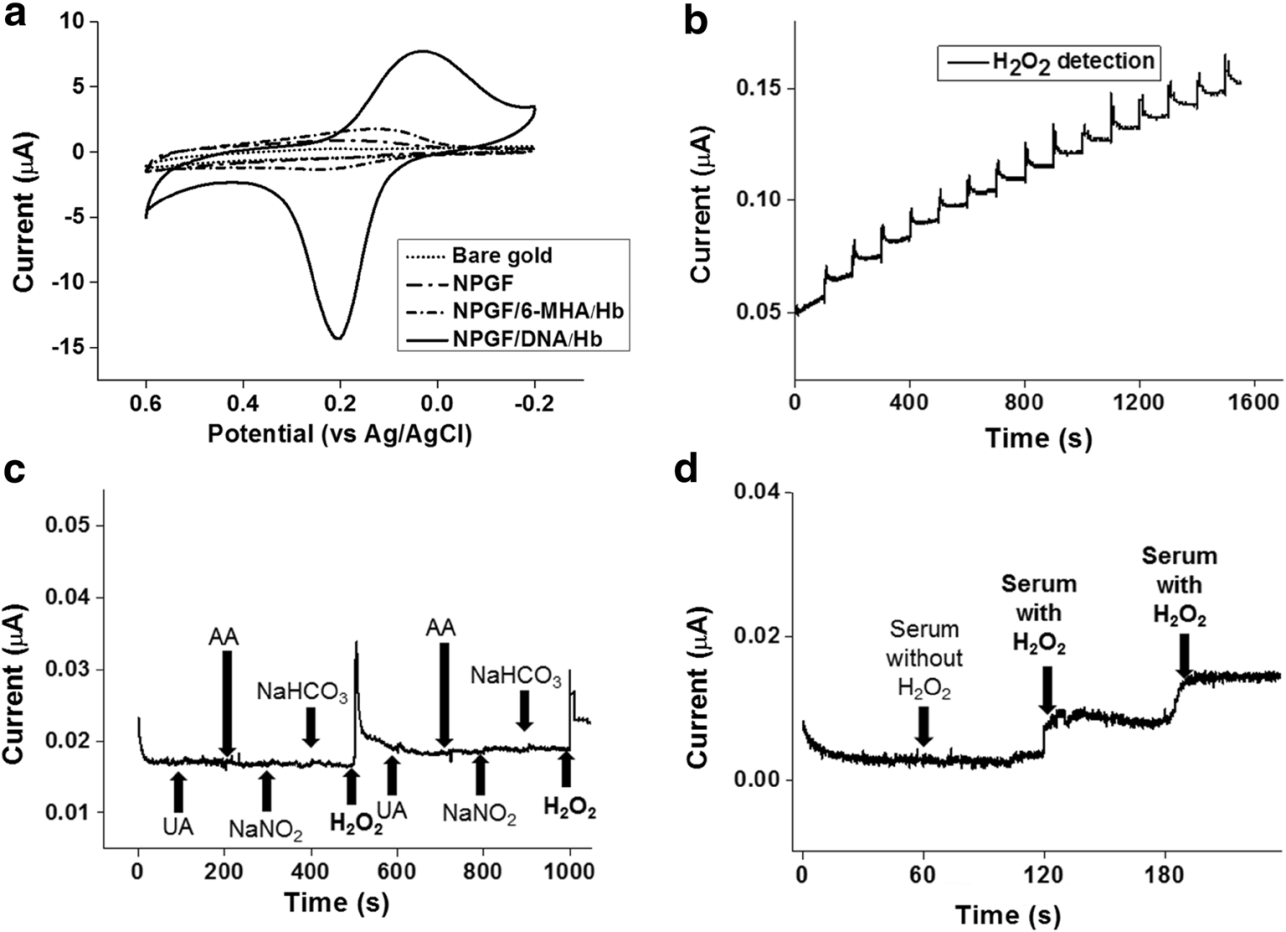
**a** Cyclic voltammograms of Bare gold, NPGF electrode, NPGF/6-MHA/Hb, NPGF/DNA/Hb in PBS; amperometric i-t curves of **b** addition of 10 μL of 100 μM H_2_O_2_ solution; **c** Successive addition of 1 µM UA, AA, NaNO_2_, NaHCO_3_, and 1 µM H_2_O_2_ solution; **d** successive addition of serum without H_2_O_2_ once and serum with 1 µM of H_2_O_2_ twice

Figure 4a illustrated that the fabricated sensor showed signal enhancement comparing to bare gold electrode, NPGF electrode, Hb on the NPGF by 6-MHA linker and Hb on the NPGF by DNA hybridization. The fabricated biosensor showed reduction peak at 0.20 V. It also showed an oxidation peak at 0.0 V. At reduction peak, HbFeIII reacted with H_2_O_2_ and electron to give HbFeII. On the other hand, HbFeII was decomposed as HbFeIII, electron and H_2_O_2_ at oxidation peak [29]. When comparing electrochemical signal between the NPGF and the bare gold, signal enhancement of the NPGF electrode was verified because of the expanded surface area by compactly deposited gold nanoparticles on the gold electrode. Figure 4a showed that the signal by DNA hybridization was improved comparing to the conventional sensor which is composed of 6-MHA which is known as chemical linker. Especially in the range of 0.10 to 0.20 V, the DNA hybridization sensor showed ten times higher reduction peak than the 6-MHA chemical linker sensor. As shown in Fig. 4a, the electrochemical signal was enhanced by the NPGF electrode and DNA hybridization. Additional file 1: Fig. S3 showed stability test with CV and showed storage stability after 25 cycles.

Fourier-transform infrared spectroscopy (FT-IR) analysis was also conducted to verify whether Hb was denatured or not at Additional file 1: Fig. S6. FT-IR spectroscopy is sensitive to the secondary structure of the protein. FT-IR analysis showed that Hb is not denatured after DNA hybridization to the cDNA. As widely known, the shapes of the amide I infrared absorbance bands of Hb provide specified information on the secondary structure of the polypeptide chain. The amide I band (1750–1650 cm^−1^) is caused by C=O stretching vibrations of peptide linkages. In our result, the amide I band of Hb on the NPGF electrode was located at 1730.29 cm^−1^, and we verified Hb is not denatured.

### Detection of H_2_O_2_ by fabricated biosensor

The amperometric i–t curve was obtained for the Hb-DNA conjugate on the NPGF electrode to measure the enzymatic reaction of the biosensor. The amperometric reaction of the biosensor was performed by continuous addition of 10 μL of 100 μM H_2_O_2_ solution.

**Fig. 5:**
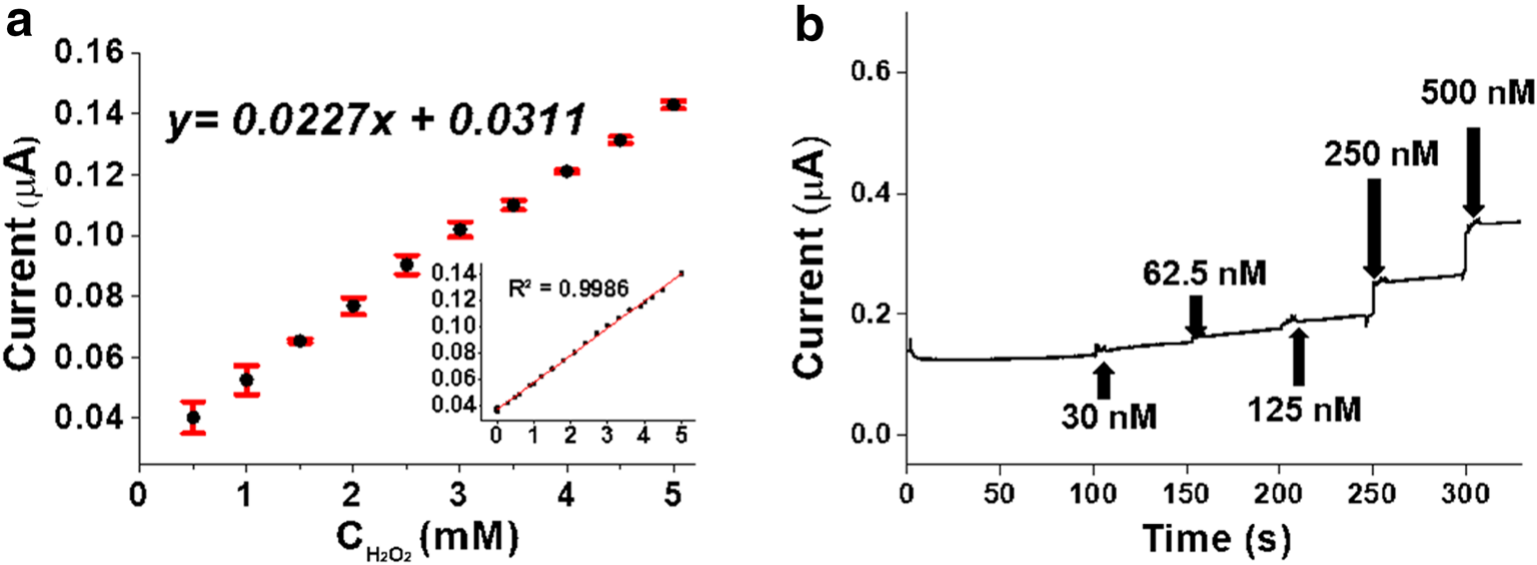
**a** Calibration curve of 0.5 mM concentration of H_2_O_2_ versus catalytic peak current and error bar with trend line equation and linear range (0.00025–5.0 mM) with a correlation coefficient of 0.9986; **b** amperometric response curves of Hb/DNA/NPGF addition of 30 nM, 62.5 nM, 125 nM, 250 nM and 500 nM H_2_O_2_ solution

To achieve the complete reduction state of the Hb before the enzymatic reaction, 0.2 V was applied as the initial potential. The amperometric reaction curve for the enzymatic reaction of the biosensor during the addition of H_2_O_2_ is shown in Fig. 5. When 100 μM H_2_O_2_ solution was added every 100 s, the reduction current of the biosensor was increased sharply with a steady amount of current (Fig. 4b).

To confirm the selectivity of the biosensor, 1 μM of UA, AA, NaNO_2_ and NaHCO_3_ were added every 100 s subsequently to observe the response current of biosensor (Fig. 4c). In the only case of 1 μM H_2_O_2_ addition, the response current was increased compared to the results of UA, AA, NaNO_2_ and NaHCO_3_ addition.

The interferential experiment was conducted with real human blood serum sample. It was conducted to check interference when the proposed sensing platform is applied at in vivo. The ratio of serum was maintained as 6% of total volume just like the real human blood. After 60 s of signal stabilization, 10 μL of serum without H_2_O_2_ was added to 5 mL of PBS electrolyte and showed no response current (Fig. 4d). To make clear, detection limit was confirmed as 250 nM at analyte with serum at Additional file 1: Fig. S5. So serum was verified as non-interferential byproduct to detect hydrogen peroxide. 100 μM of H_2_O_2_ solution were added twice after 60 s of stabilization, two stable response current were observed. These outstanding performance of our proposed sensor can be applied as a sensing platform in the field of in vivo biosensor at the future.

The reproducibility and repeatability of the proposed sensor was also studied by amperometric response with 1 mM H_2_O_2_ at PBS buffer solution. Additional file 1: Fig. S4 showed response current from three equally fabricated individual sensors. The relative standard deviation (R.S.D.) from three sensors showed 5.6%. Also, one of uniformly fabricated sensor showed repeated response current at same concentration H_2_O_2_ during 10 successive addition and the R.S.D. showed 6.8%. Also, from the trend line equation (Y = 0.0227x + 0.0311), we can assume that it showed wide linear range (0.00025–5.0 mM) with little error bar from three individually fabricated sensors with 10 measurements (Fig. 5a). From the results, we could conclude fabricated sensor showed similar performance respectively.

To test the detection limit of the biosensor, various concentrations of H_2_O_2_ (30, 62.5, 125, 250 and 500 nM) were added to the sensor (Fig. 5b). According to the amperometric results, the detection limit of the biosensor for H_2_O_2_ showed as 250 nM. And it also showed remarkable performance comparing other enzymatic sensors. So various types of H_2_O_2_ sensors were compared to prove the outstanding performance of our work (Additional file 1: Table S1).

## Conclusions

In the present study, an electrochemical biosensor composed of Hb-DNA conjugate on the NPGF was fabricated for H_2_O_2_ detection with electrochemical signal enhancement and selectivity. The Hb-DNA conjugate was fabricated to immobilize the Hb-DNA conjugate uniformly onto the electrode without Hb aggregation. Furthermore, the NPGF electrode fabricated by electrodeposition technique was used to extend the surface area of the electrode for electrochemical signal enhancement. The fabricated Hb-DNA conjugate was verified by UV–VIS and SDS-PAGE. The fabrication of the NPGF electrode by electrodeposition was confirmed by SEM. The preparation of the biosensor by DNA hybridization was also investigated by CV. This biosensor prepared with DNA and NPGF electrode showed the uniform orientation of sensing molecule on the electrode compared to electrode fabricated without DNA hybridization. The electrochemical properties of this biosensor showed the enhanced electrochemical signal compared to the conventional electrode prepared without NPGF electrode. Moreover, the proposed biosensor composed of Hb-DNA conjugate on the NPGF electrode showed selective amperometric response with selective performance for detecting H_2_O_2_ in mixtures added with NaHCO_3_ and AA. In the result, the proposed biosensor composed of Hb-DNA conjugate on the NPGF electrode can be used as a powerful sensing platform for biosensor development with electrochemical signal enhancement and high selectivity.

## Additional file

**Additional file 1: Figure S1.** SEM images of electrodeposition under A) −1.5 V and 30 s; B) −1.3 V and 45 s. **Figure S2.** Column diagram of the NPGF nanoparticle size with standard error values. **Figure S3.** Cyclic voltammograms of 1st cycle and 25th cycle. **Figure S4.** Column diagram of three individually fabricated sensors with standard error values. **Figure S5.** Amperometric response curves of addition of 125 nM, 250 nM and 500 nM H_2_O_2_ solution with serum analyte. **Figure S6.** FT-IR analysis of A) NPGF/cDNA; B) NPGF/DNA/Hb. **Table S1.** Comparison of several H_2_O_2_ sensors.

## Abbreviations

HRP: horseradish peroxidase
NPGF: nanoporous gold thin film
Sulfo-SMCC: sulfosuccinimidyl 4-cyclohexane-1-carboxylate
PEG: polyethylene glycol
TCEP: tris(2-carboxyethyl)phosphine
TE: tris-EDTA
DTT: dithiothreitol
6-MHA: 6-mercaptohexanoic acid
RA: roughness average
Rq: RMS roughness
Rmax: maximum roughness
