# Supplementary material for: H2O2 biosensor consisted of hemoglobin-DNA conjugate on nanoporous gold thin film electrode with electrochemical signal enhancement
Source: Nano Converg. 2019 Jan 3;6:1. doi: 10.1186/s40580-018-0172-z (PMC6314933; doi:10.1186/s40580-018-0172-z)
Supplement: Supplementary file 1 — Additional file 1: Figure S1. SEM images of electrodeposition under A) −1.5 V and 30 s; B) −1.3 V and 45 s. Figure S2. Column diagram of the NPGF nanoparticle size with standard error values. Figure S3. Cyclic voltammograms of 1st cycle and 25th cycle. Figure S4. Column diagram of three individually fabricated sensors with standard error values. Figure S5. Amperometric response curves of addition of 125 nM, 250 nM and 500 nM H2O2 solution with serum analyte. Figure S6. FT-IR analysis of A) NPGF/cDNA; B) NPGF/DNA/Hb. Table S1. Comparison of several H2O2 sensors. [file 40580_2018_172_MOESM1_ESM.docx]

**SUPPLEMENTARY INFORMATION TO NANO CONVERGENCE**

Title: H_2_O_2_ Biosensor Consisted of Hemoglobin-DNA Conjugate on Nanoporous Gold thin Film Electrode with Electrochemical Signal Enhancement

**
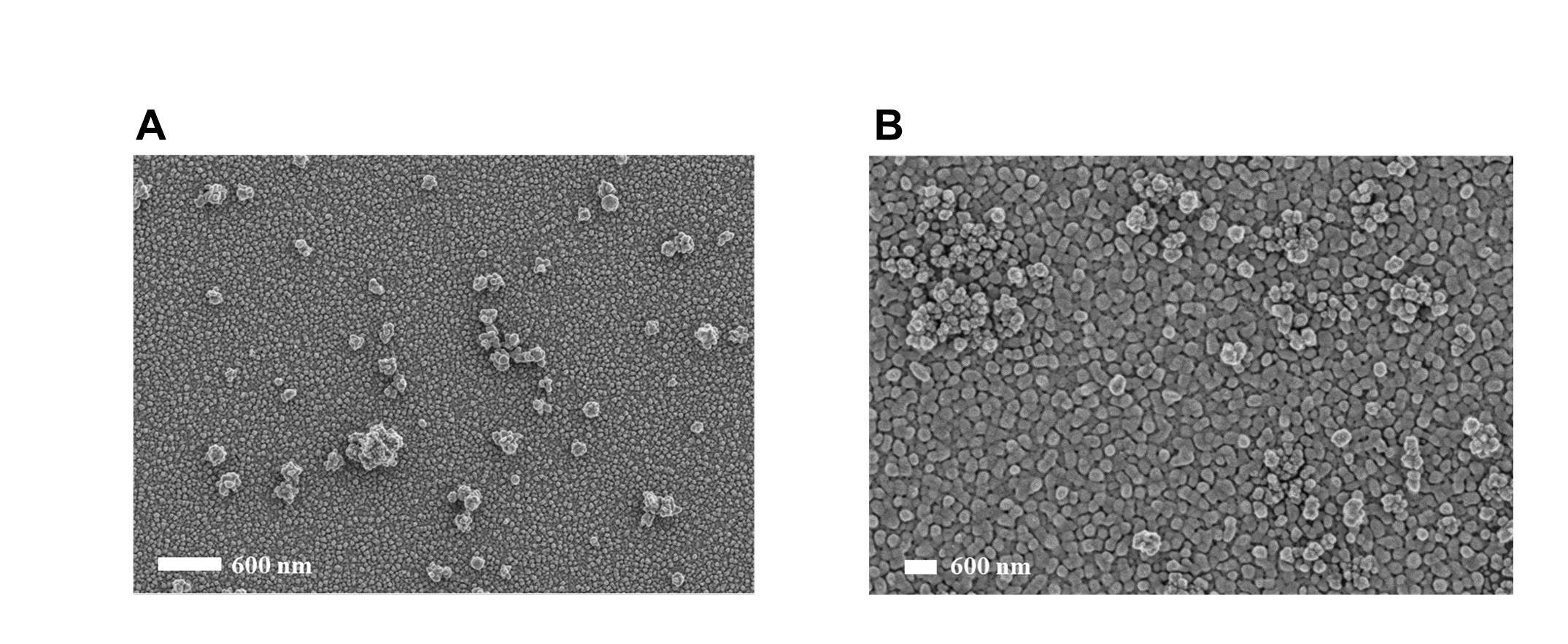
**

**Figure S1.** SEM images of electrodeposition under A) -1.5 V and 30 s; B) -1.3 V and 45 s**.**

**
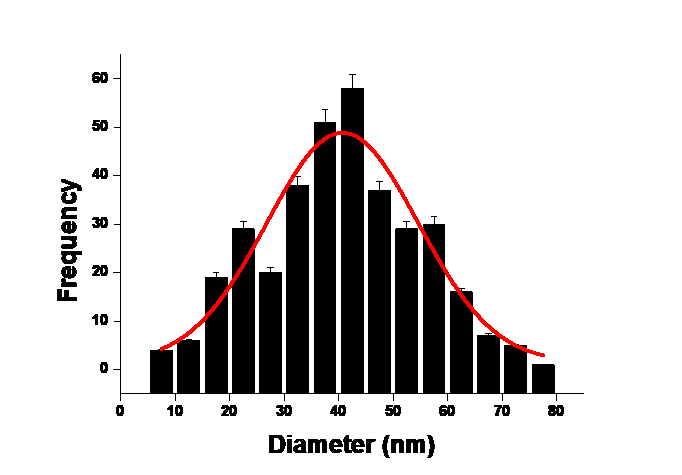
**

**Figure S2.** Column diagram of the NPGF nanoparticle size with standard error values.

**
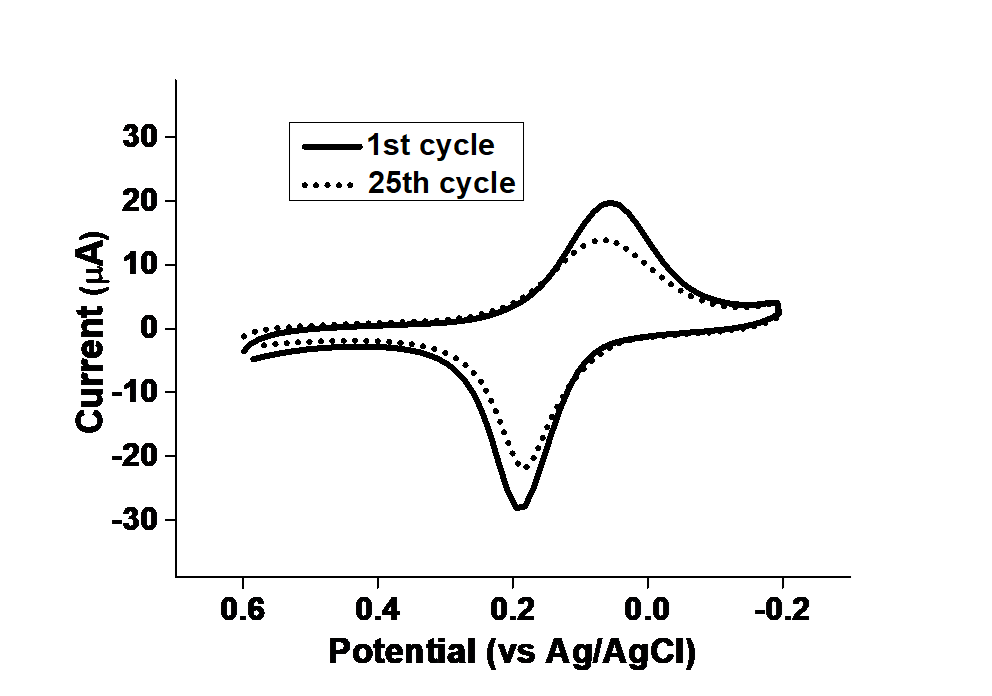
**

**Figure S3.** Cyclic voltammograms of 1st cycle and 25th cycle.

**
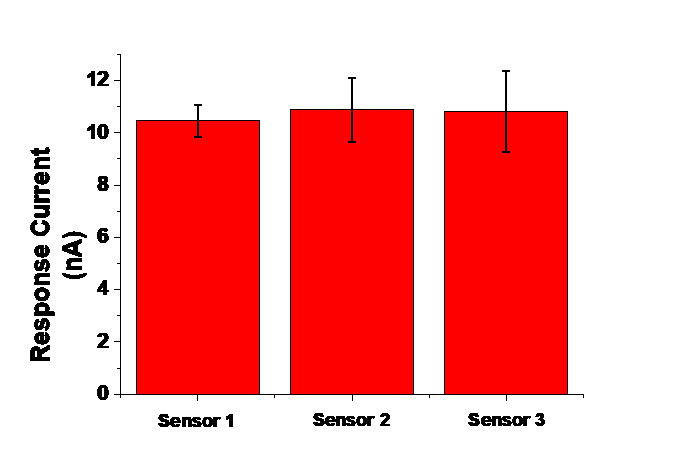
**

**Figure S4.** Column diagram of three individually fabricated sensors with standard error values.

**
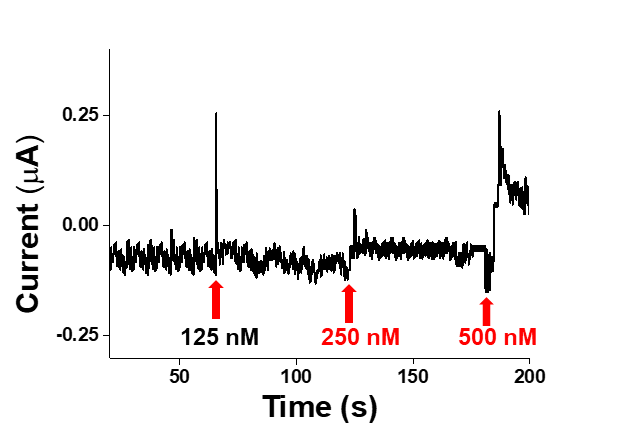
**

**Figure S5.** Amperometric response curves of addition of 125 nM, 250 nM and 500 nM
H_2_O_2_ solution with serum analyte.

**
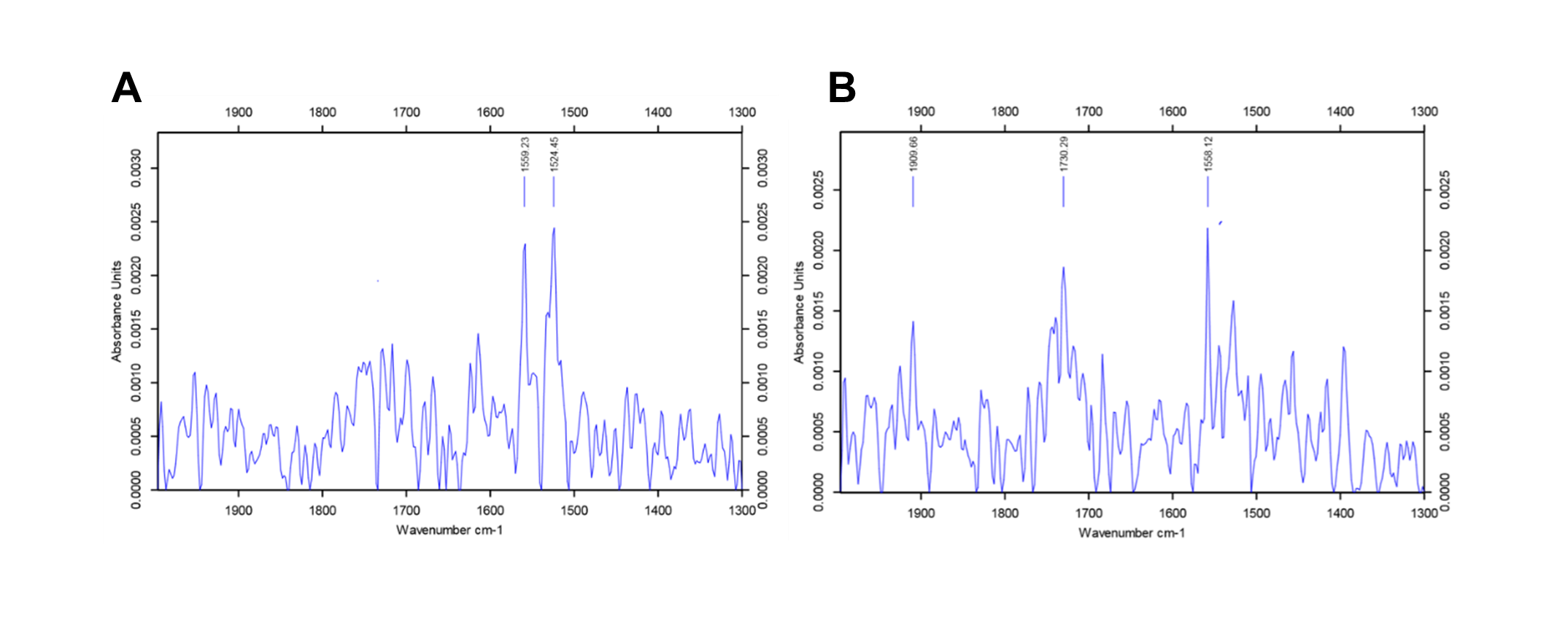
**

**Figure S6.** FT-IR analysis of A) NPGF/cDNA; B) NPGF/DNA/Hb.

**Table S1.** Comparison of several H_2_O_2_ sensors

| **Types of Electrode** | **Linear Range**  **(mM)** | **Detection Limit**  **(μM)** | **Reference** |
| --- | --- | --- | --- |
| Hb/SA-MWCNTs/GCE | 0.04 - 0.20 | 16.4 | [1] |
| Hb/CNTPME | 0.21 - 0.90 | 9.0 | [2] |
| MnO_2_ /carbon fiber microelectrode | 0.01 - 0.26 | 5.4 | [3] |
| MWCNT@Hb/Nf^c^ | 0.05 - 0.50 | 2.4 | [4] |
| Hb/Graphene/CS | 0.01 - 0.23 | 0.51 | [5] |
| Hb/DNA/NPGF | 0.00025 - 5.00 | 0.25 | **This work** |

[1] H.Y. Zhao, W. Zheng, Z.X. Meng, H.M. Zhou, X.X. Xu, Z. Li, Y.F. Zheng, Biosens. Bioelectron. **24**, 2352–2357 (2009)

[2] Y.D. Zhao, Y.H. Bi, W.D. Zhang, Q.M. Luo, Talanta **65**, 489-494 (2005)

[3] S.B. Hocevar, B. Ogorevc, K. Schachl, K. Kalcher, Electroanalysis. **16**, 1711–1716 (2004)

[4] A.S. Kumar, P. Gayathri, P. Barathi, R. Vijayaraghavan, J. Phys. Chem. C. **116**, 23692–23703 (2012)

[5] H. Xu, H. Dai, G. Chen, Talanta **81**, 334–338 (2010)
